# Effects of Nonpharmacological Interventions on Balance Function in Patients with Osteoporosis or Osteopenia: A Network Meta-Analysis of Randomized Controlled Trials

**DOI:** 10.1155/2021/6662510

**Published:** 2021-05-04

**Authors:** Lu Zhu, Wenzhong Wu, Ming Chen, Daoming Xu, Huaning Xu, Lanying Liu, Jing Liu, Zequan Zhu

**Affiliations:** Affiliated Hospital of Nanjing University of Chinese Medicine, Nanjing 210029, Jiangsu, China

## Abstract

**Objective:**

To evaluate the clinical efficacy of nonpharmacological interventions in improving balance function of patients with osteoporosis or osteopenia using network meta-analysis (NMA).

**Methods:**

We searched seven databases (PubMed, the Cochrane Library, Embase, CKNI, Wanfang Data, VIP, and CBM) for relevant randomized controlled trials (RCTs) up to August 31, 2020. Berg Balance Scale (BBS) and Time Up and Go Test (TUGT) were used as outcome measures. Two researchers independently screened studies, collected data from the studies, and estimated risk of study bias. Divergence in the evaluation process was settled by consulting a third researcher. We used Stata 15.1 software for network meta-analysis (NMA).

**Results:**

We identified 10 eligible RCTs, with a total of 737 patients and four intervention methods, including traditional Chinese medicine (TCM) exercises, pulsed electromagnetic fields (PEMFs), strength training, and balance and strength training. The results revealed that all nonpharmacological interventions could improve balance function, but the effect of balance and strength training was better than other interventions.

**Conclusion:**

Exercise can significantly improve the balance function of patients with osteoporosis and osteopenia, and balance combined with strength training has the best effect, followed by TCM exercises.

## 1. Introduction

Osteoporosis is characterized by deterioration of bone microstructure and loss of bone mass and easily leads to fracture. Osteopenia is the precursor of osteoporosis. The fracture rate of women with osteoporosis is 2.7 times higher than that of average women, and the fracture rate of women with osteopenia is 1.7 times higher than that of average women [1]. Antiosteoporosis drug treatment has become increasingly mature. However, due to media overreporting of rare adverse reactions to related drugs and the tendency of high-risk groups for noncompliance, the prevalence of fracture rates continues to increase [2]. Fractures can lead to further deterioration of physical function, malnutrition, depression, and even death; thus, the impacts on individuals, the medical system, and the whole community cannot be ignored [3–5]. The prevention of fracture in high-risk groups has, therefore, become an important challenge in global health.

Fall risk is an independent factor affecting fracture frequency, and decline in balance ability is highly correlated with the incidence of falls [6]. Current research confirms that the Berg Balance Scale (BBS) of people with a history of falls is significantly lower than that of people without a history of falls [7, 8]. At present, effective drugs such as bisphosphonates, teriparatide, and estrogen are recommended for the treatment of bone loss prevention. Although some studies have shown these drugs can increase bone mineral density (BMD) and improve balance thereby preventing falls [9], results are not consistent. Extensive development of nonpharmacological interventions suggests promise for the prevention of fracture [2, 10, 11]. Thus, we set out to conduct a network meta-analysis of nonpharmacological interventions; specifically, we consider acupoint stimulation therapy, traditional Chinese medicine (TCM) exercises, kinesitherapy, occupational therapy, and physiotherapy. We studied the effects on balance function of these therapies in patients with osteoporosis and osteopenia to help doctors develop better treatment strategies.

## 2. Materials and Methods

### 2.1. Design and Registration

Research was conducted in accordance with the Preferred Reporting Items for Systematic Review and Meta Analyses (PRISMA) statement (http://www.prismatatement.org/) and is registered in the PROSPERO database as CRD42020206838.

### 2.2. Search Strategy

We searched studies on PubMed, the Cochrane Library, Embase, CKNI, Wanfang Data, VIP, and CBM databases for randomized controlled trials (RCT) up to August 31, 2020, based on MESH terms and free words, and pertinent details of results saved.

### 2.3. Inclusion and Exclusion Criteria

#### 2.3.1. Inclusion Criteria

*(1) Types of Patients*. The 1994 diagnostic criteria recommended by the WHO defines those with a BMD value lower than the peak bone value of normal adults of the same gender and race by 1–2.5 standard deviations as having osteopenia, and those with a reduction ≥2.5 standard deviations as having osteoporosis; individuals meeting either criteria were included.

*(2) Interventions*. Nonpharmacological interventions included in the experimental group were acupoint stimulation therapy, TCM exercises, kinesitherapy, occupational therapy, and physiotherapy. The control group included the basic treatment recommended by national guidelines [12] (supplemental calcium and vitamins), nonintervention (waiting for treatment), sham acupuncture therapy, or placebo.

#### 2.3.2. Exclusion Criteria

1. The study language was not Chinese or English
2. Multiple articles using data from the same study
3. Studies with only summary data where we were unable to get required full data

### 2.4. Outcome Measures

#### 2.4.1. Berg Balance Scale (BBS) [13]

This includes a static and dynamic balance function assessment, involving tasks from daily life such as sitting up, transfer, and other aspects. The scale has high reliability and good validity, and globally, BBS has become the most widely used balance scale in clinical practice.

#### 2.4.2. Time Up and Go Test (TUGT) [14]

This test is used to measure balance ability during functional activities such as sitting up and walking, to predict fall risk. It is widely used in the evaluation of neurological and skeletal diseases.

### 2.5. Study Selection and Data Extraction

Two of the authors (L. Zhu and M. Chen) individually selected studies and collected data, importing the identified studies into EndNote X9.0. The first step in studies selection was to exclude published studies that used the same data. In the next stop, after reading the title and abstract, any nonrelevant studies were excluded. The remaining studies were then read in detail to determine final inclusion. The researcher used a Microsoft Excel spreadsheet to input the following data from each included study: author names, publishing date, diagnosis criteria, specific information of the experimental and control groups, outcome measures after intervention, results, and risk assessment of bias. Disagreements between L. Zhu and M. Chen were settled by a third author (D. M. Xu).

### 2.6. Assessing the Risk of Bias

To evaluate the risk of bias in the 10 RCT studies included, the Cochrane Collaboration tool [15] was used. The assessment criteria consist of seven items: random sequence generation, allocation concealment, blinding of participants and personnel, blinding of outcome assessment, incomplete outcome data, selective reporting, and any other biases. Criteria score disagreements of studies between the two reviewers were resolved by the third reviewer (D.M. Xu). The assessment result was generated using Review Manager 5.3.

### 2.7. Data Synthesis and Analysis

The meta/mvmeta/network data package for the software Stata 15.1 was used for network meta-analysis (NMA). Measurement data were converted to standard mean differences (SMD) as the effect analysis statistic, along with 95% confidence interval (95% Cl) of the SMD. The mvmeta installation package in Stata was used to process the data, and a network diagram, meta-analysis diagram (prediction interval diagram), and effectiveness order diagram were drawn. Where no statistically significant difference (*P* > 0.05) was present, the consistency model was used to analyze and rank the results. Otherwise, the nonuniform model was used for analysis of statistically significant differences. The surface under the cumulative ranking curve (SUCRA) was used to assess the likelihood of each intervention being the best intervention. An absolute SUCRA value between zero and one indicated that the intervention was effective, while a zero indicated the intervention was ineffective. Intervention measures were ranked according to the value of SUCRA.

## 3. Results

### 3.1. Studies Searching

**Figure 1:**
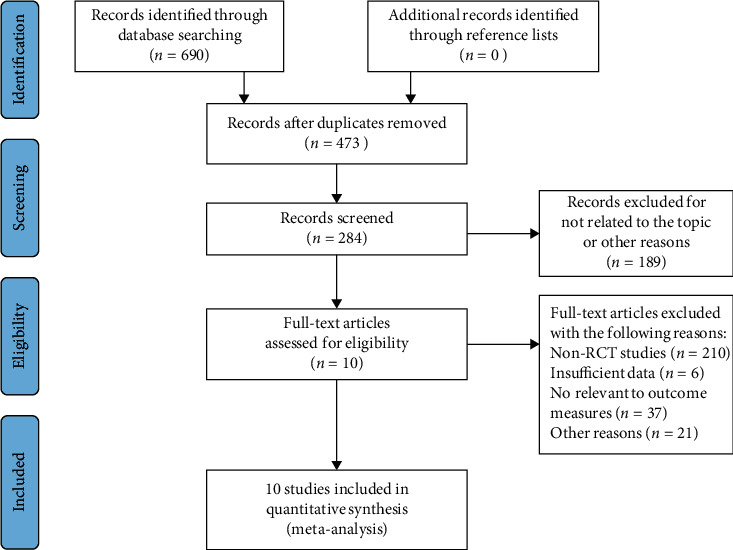
PRISMA flow chart of search results.

The initial screening found 690 studies; 10 RCTs were included in the NMA after applying exclusion and inclusion criteria.  Figure 1 shows a flow chart of this process.

### 3.2. Characteristics of the Identified Studies

Among the 10 identified RCTs, three studies were in English [16–18] and seven in Chinese [19–25]. Publication year ranged from 2009 [17] to 2019 [21], and all studies were two-arm trials. A total of 737 patients aged 50–83 years were included, and the sample size of studies ranged from 43 to 111 and treatment period ranged from 1–12 months. Two diagnostic and therapeutic evaluation criteria were used, including the 1994 WHO guidelines and 2011 primary osteoporosis guidelines. The 10 RCTs included five intervention methods: TCM exercises, pulsed electromagnetic fields (PEMFs), strength training, balance and strength training, and basic treatment (it needs to be explained here, strength training, balance and strength training belong to kinesitherapy, PEMFs belongs to physiotherapy, and no eligible occupational therapy RCT has been found).Characteristics of the included studies are shown in Table 1.

### 3.3. Risk of Bias of the Individual Studies

**Figure 2:**
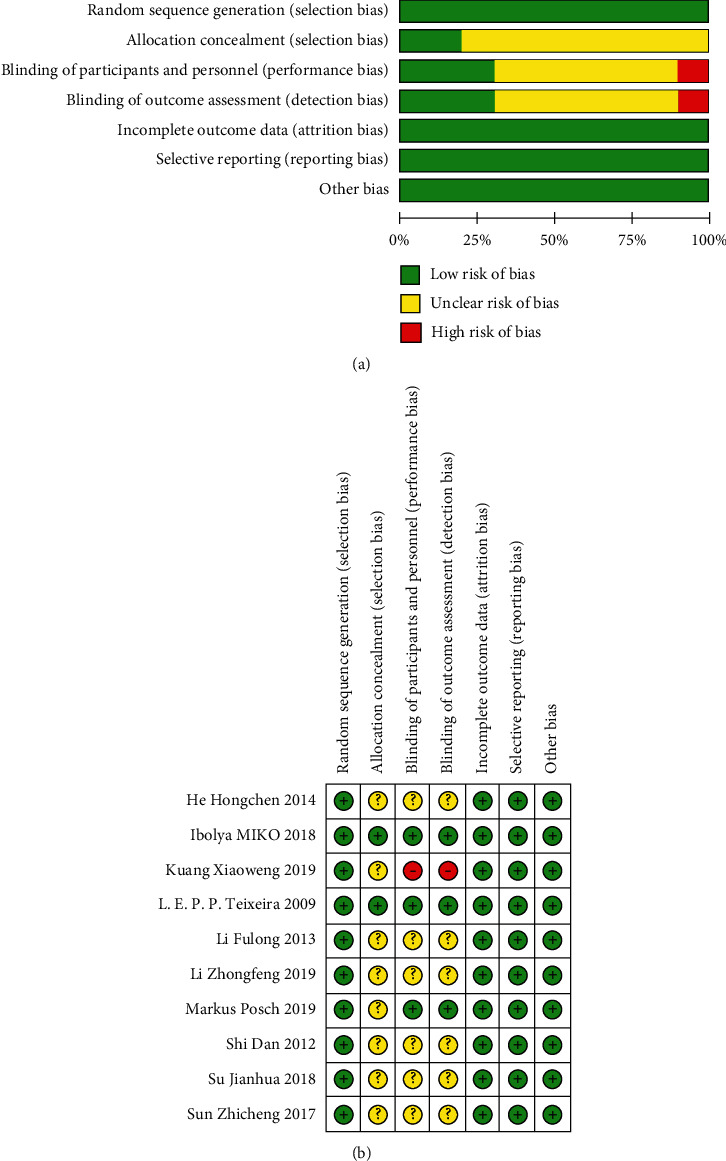
Risks of bias graph and summary for included RCTs. The left (risk of bias graph) shows an overall risk of bias of each domain. The right (risk of bias summary) indicates the risk of bias of each domain in each study.

All the identified studies reported randomization. Eight studies specifically described adequate random sequence generation methods, including envelope method and random number table [16–20, 22–24]. Given the particularity of nonpharmacological interventions, it was extremely difficult to blind both the patients and the personnel to treatment, and only three studies described sufficient allocation concealment and the specific implementation methods for blind evaluation of outcome measures [16–18]. Six studies reported loss of follow-up during treatment, explaining the number of lost cases and specific reasons [16–19, 22, 23]. One study reported adverse reactions [17]. The overall quality of the identified studies was acceptable.  Figure 2 shows the risk of bias generated by Review Manager 5.3.

### 3.4. Network Meta-Analysis of Outcome Measures

#### 3.4.1. Network Plot

**Figure 3:**
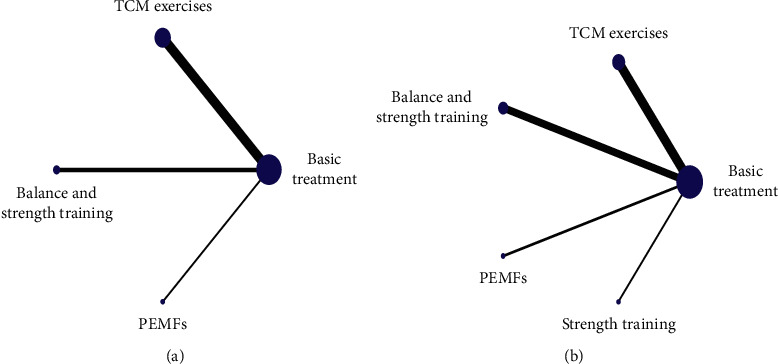
Network plot of comparing the efficacy on balance function of various interventions. (a) BBS. (b) TUGT.

Figure 3 is the network plot of this study. The size of the node represents the study sample size. The line between the nodes indicates the two linked interventions are directly comparable; no line indicates that there is no evidence for direct comparison. Thickness of lines is positively correlated with the quantity of direct comparison studies.

#### 3.4.2. BBS

**Figure 4:**
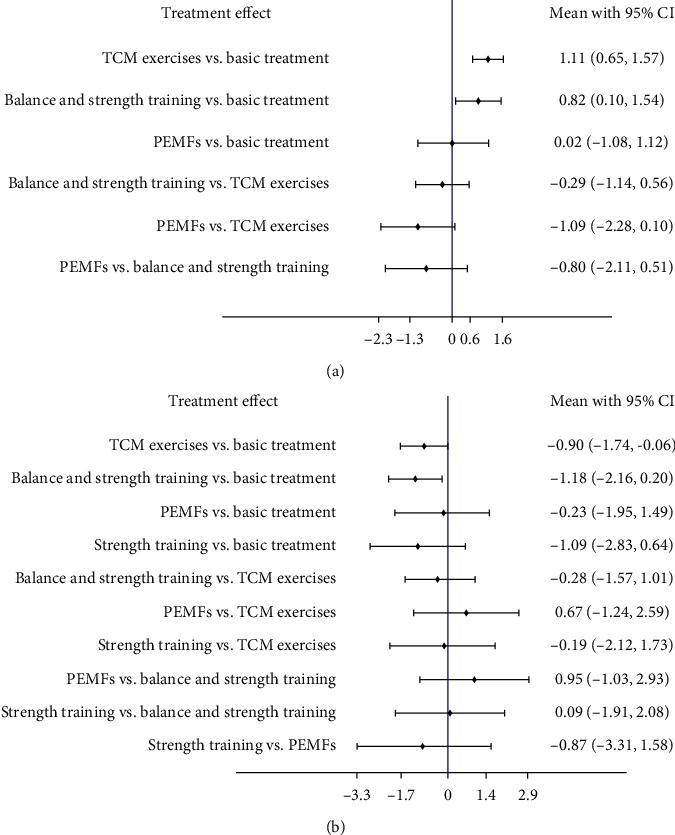
The results of network meta-analysis. (a) BBS. (b) TUGT.

Three interventions are related to BBS, including PEMFs, balance and strength training, and TCM exercises. The network diagram of BBS (Figure 3(a)) indicates that the number of studies comparing TCM exercises with basic treatment is greatest that the number of studies comparing the other two interventions with basic treatment is small, and there is no evidence of indirect comparison among the three interventions. Based on NMA results, TCM exercises (SMD = 1.11; 95% CI (−0.65, 1.57)), balance and strength training (SMD = 0.82; 95% CI (0.10, 1.54)) were better than basic treatment, and PEMFs (SMD = 0.02; 95% CI (−1.08, 1.12)) was similar to basic treatment (Figure 4(a)).

#### 3.4.3. TUGT

Four interventions were related to TUGT: TCM exercises, balance and strength training, strength training, and PEMFs. A network diagram of TUGT (Figure 3(b)) indicates that, compared with basic treatment, the number of studies on TCM exercises is greatest, followed by studies of balance and strength training. Fewer comparisons exist between the other two interventions and basic treatment, and there is no evidence of indirect comparison among the four interventions. The NMA results reveal that balance and strength training (SMD = −1.09; 95% CI (−2.83, 0.64)), strength training (SMD = −1.18; 95% CI (−2.16, −0.20)), TCM exercises (SMD = −0.90, 95% CI (−1.74, −0.06)), and PEMFs (SMD = −0.23, 95% CI (−1.95, 1.49)) were superior to basic treatment (Figure 4(b)).

### 3.5. Ranking

#### 3.5.1. BBS

**Figure 5:**
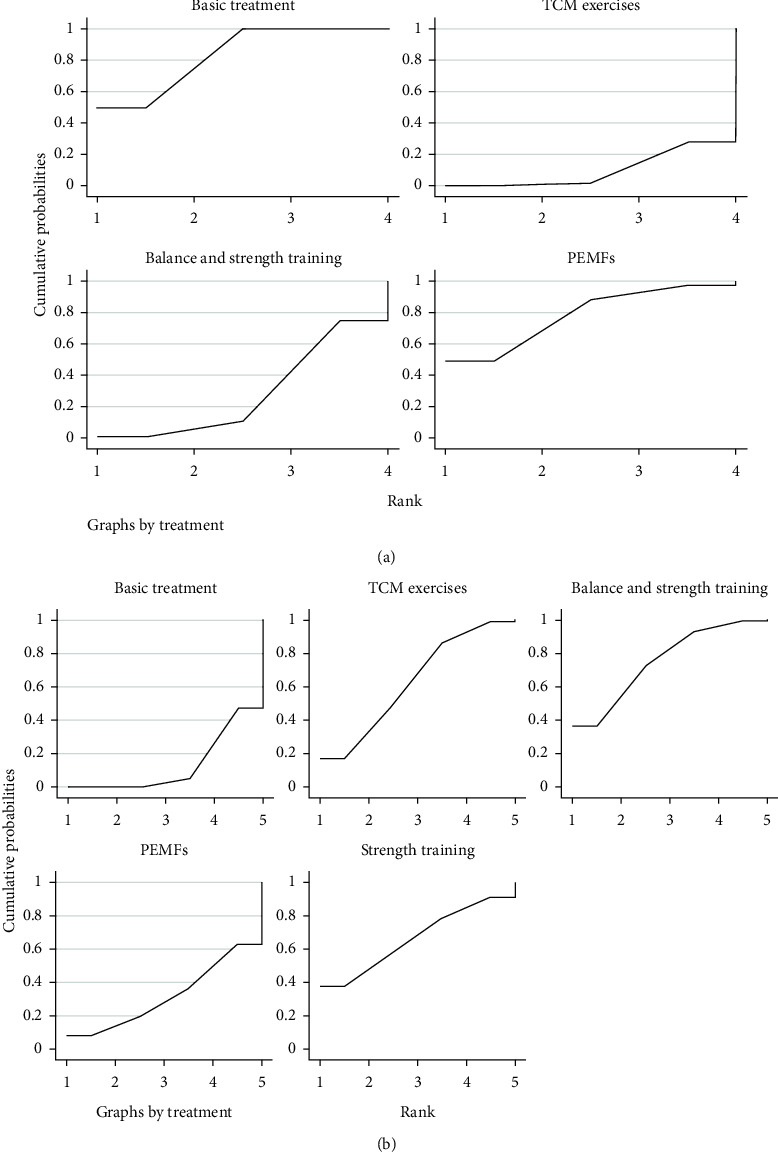
SUCRA for improving balance function of patients with osteoporosis or osteopenia. (a) BBS. (b) TUGT.

The balance function was positively correlated with BBS. Therefore, the higher the ranking of SUCRA, the worse the effect. The results rank the treatments from best to worst as TCM exercises (SUCRA = 9.8%), balance and strength training (SUCRA = 28.8%), PEMFs (SUCRA = 78.3%), and basic treatment (SUCRA = 83.1%) (Figure 5(a)).

#### 3.5.2. TUGT

TUGT and balance function are negatively correlated, so that a lower SUCA rating is worse. The best-to-worst ranking order for TUGT is balance and strength training (SUCRA = 75.3%), strength training (SUCRA = 66.6%), TCM exercises (SUCRA = 62.9%), PEMFs (SUCRA = 32.0%), and basic treatment (SUCRA = 13.1%) (Figure 5(b)).

#### 3.5.3. Syntghetical Arrangement

The relative ranking results of the two outcome indicators were not completely consistent. Overall, the effect of balance and strength training appears to be the best intervention at present.

### 3.6. Sensitivity Analysis

**Figure 6:**
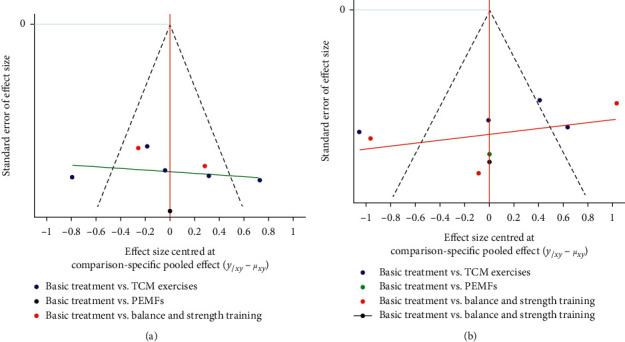
Funnel plots for RCTs included in this study. (a) BBS. (b) TUGT.

The funnel plot indicates studies were evenly distributed along both sides of the funnel, but the distribution was slightly asymmetrical, indicating that there was some publication bias and small sample size effect. (Figure 6).

## 4. Discussion

Our results show that, among the five selected nonpharmacological intervention methods, balance and strength training had the best effect, followed by TCM exercises. This conclusion is consistent with part of the latest nonpharmacological intervention clinical practice guidelines for osteoporosis treatment [26].

Balance function in the human body depends on reconciliation of information from vision, proprioception, and vestibular sensations and control of the motion effector by the central nervous system. Among these, proprioception has a great influence on the balance function of osteoporosis patients [27, 28]. Balance training is an important means of proprioception reconstruction. Stimulating proprioceptive receptors through balance training allows humans to respond more quickly, which is essential to preventing falls [29]. Madureira et al. [30] researched women with osteoporosis, with the intervention group taking a balance training program for 12 months. A considerable difference occurred between the intervention group and the nontraining group in terms of BBS (5.5 ± 5.67 vs. −0.5 ± 4.88, *P* < 0.001). Similarly, the fall frequency in the training group was lower than that of the nontraining group (−0.77 ± 1.76 vs. 0.03 ± 0.98, *P*=0.018). Another recent meta-analysis [31] also confirmed that balance training was significantly associated with a decreased frequency of falls in osteoporosis patients.

Proprioceptive receptors exist in joints and tendons, among other areas of the body, and the integration of the nerve and musculoskeletal systems can promote recovery of proprioception [32]. Suri et al. [33] found that trunk muscle strength affected balance ability in an elderly community (mean age = 76 years) and inferred that improving trunk muscle strength may be an important approach to maintaining balance and minimizing risk. There are various forms of muscle strength training, and each training method contains different components, operation process, load requirements, and suitable population. In recent years, the studies on vibration training and core muscle strength training at home and abroad are relatively prominent [34]. Vibration training can make low-intensity mechanical stimulation signals act on bones and muscles with high frequency. Li et al. [35] found that whole-body vibration training can improve the coordination of the limbs through the adjustment of the musculoskeletal, compared with the simple squat movement, it can obviously improve the balance function. Core muscle strength training creates a dynamic training environment with the help of a dynamically unstable support surface. With a changing center of gravity position, the body must constantly adjust to control the gravity center and maintain balance and stability in posture; such training improves proprioception function [36–38]. By increasing the stability of the core area, the muscle dynamic chain power transmission further regulates the upper and lower limbs to enhance the overall balance ability of the human body [38, 39].The clinical trial of Teixeira et al. [17], included in this study, found that, compared with patients only receiving antiosteoporosis drugs, the maximal dynamic strength of the quadricep muscles in osteoporosis patients receiving balance training and lower limb muscle strength training, increased by 76% on average, and that TUGT scores in the training group also improved (*P* < 0.0001). At a 6 month follow-up, falls in the training group were significantly lower than those in the medicine group (IRR = 0.263, 95% CI (0.10–0.68), *P*=0.0064). In addition to lower limb muscle strength training, core muscle training has been recognized as an important part of strength training in recent years. Miko et al. [18] designed a complex exercise training program consisting of balance and strength training for core muscle groups (transverse abdominis muscle and multifidus muscle), and clinical trial results showed that, after one year, significant differences in TUGT and BBS scores existed in a between-group comparison (*P* < 0.005). Although the benefits of training in fall prevention and fractures are recognized, the patient's compliance needs to be considered. Although the benefits of training in fall prevention and fractures are recognized, the patient's compliance must be considered. Increasing numbers of studies [40, 41] have found that short-term intensive multimode exercises training are more conducive to implementing improved skeletal-muscle health than long-term single exercise training. Considering the compliance of patients, balance and strength training is currently the best exercise program to improve balance function for people with abnormal bone mass.

In the results of our study, TCM exercise ranked second, generally speaking, it has its own advantages. Guided by the theory of Yin-Yang and five elements, TCM exercises promote the circulation of viscera, essence, and Qi and achieve harmonization between soma and spirit. It mainly exercises the general muscle group, moves all joints, and cooperates with the rotation and winding activities of the trunk, so as to improve the flexibility and the balance ability of limbs. Existing studies have found that TCM exercises affect blood lipid, blood glucose, and hormone levels. Studies [42, 43] confirmed that “Baduanjin” can promote the hydrolysis of triglyceride by lipolipase and the synthesis of high-density lipoprotein cholesterol (HDL-C) by increasing the molecular mass of low density lipoprotein receptor (LDL-R), increasing the gene transcription and protein expression of LDL-R in liver. Also, TCM exercises can enhance the sensitivity of insulin. LAN C's study [44] on “Tai chi” also confirmed that it has the similar effects. Skeletal muscle cells contain insulin receptors, a large number of accumulation of blood lipids will increase insulin resistance, lead to hyperglycemia, and affect muscle quality. Therefore, people who exercise regularly have lower blood lipid content and stronger ability of glucose metabolism, which can improve the quality of skeletal muscle, maintain the balance function of human body, and prevent fractures.

Our NMA has some limitations and advantages, reflected in the following aspects. First, the meta-analysis study had a relatively small number of studies; the exercise time, frequency, and intensity differed among the included studies; and the dosage used for the basic treatment was not completely consistent, which may have increased heterogeneity of the studies. Therefore, to improve the reliability of our results, it will be necessary to run more clinical studies to expand sample size. Second, the risk of bias related to randomization, allocation, concealment, and blinding in most RCTs was undefined. Third, several different subtypes of osteoporosis, such as postmenopausal high conversion osteoporosis, senile low transformation osteoporosis, idiopathic osteoporosis, and secondary osteoporosis occur, and these were not distinguished in this study. In addition, this study also did not differentiate according to the severity of the disease.

Despite its limitations, this NMA is the first to assess and evaluate the clinical efficacy of nonpharmacological interventions in improving balance function of patients with osteoporosis or osteopenia. The current evidence shows that compared with routine nursing and nonintervention groups, multimode exercises training is definitely effective in ameliorating balance and promoting the recovery of muscle function and preventing tumble down. In addition, we provide sufficient evidence to show that exercise can also affect long-term bone repair through overall regulation: (1) regulating the level of hormones [45]: exercise can increase the release of estrogen, androgen, and growth hormone, thus accelerate the bone formation, also inhibit the release of parathyroid hormone, calcitonin, glucocorticoid, and other bone resorption-related hormones, and thus reduce osteoclast osteolysis. (2) Promoting calcium absorption and utilization: during exercise, the increase of blood flow in the bone cortex is conducive to the transfer of blood calcium to the bone and improve the threshold of calcium demand in the body. (3) Promoting glucose metabolism [46, 47]: exercise can improve the utilization rate of glucose, enhance the oxidative decomposition of sugar, reduce the load of islet cells, and improve the body environment. Relevant studies show that the increase of blood glucose will lead to the decline of bone matrix conversion and the loss of calcium salt and increase the incidence rate of osteoporosis. At the same time, it will also hinder the reabsorption of calcium, phosphorus, and magnesium in renal tubules, leading to secondary hyperparathyroidism and promoting osteoclasty. (4) Promoting blood lipid metabolism [48, 49]: body fat and bone mass formation present different correlation under different body fat ratio. Previous studies believe that weight gain imposes greater mechanical load on the bone to promote osteogenesis, but the current data show that the individuals with body mass index above 40 kg/m^2^ have an increased risk of osteoporosis because visceral fat energy in obese people can promote systemic inflammation and lead to bone loss. At the same time, the increase of intramuscular fat content leads to poor muscle function and increases the risk of falls. And through exercise, it can promote energy consumption, effectively increase the content of HDL-C, reduce TC and LDL-C, reduce body fat content, and the obese people can benefit from it.

## 5. Conclusion

The results of this NMA show that exercise can significantly improve the balance function of patients with osteoporosis and osteopenia, and balance combined with strength training has the best effect, followed by TCM exercise. In addition, multicenter and high-quality randomized controlled trials should be carried out to provide more details [50].

## Figures and Tables

**Table 1:** Characteristics of literatures on nonpharmacological interventions on balance function in patients with osteoporosis or osteopenia.

Study	Country	Definition	Sample size	Course of treatment	Age/course of disease (EG/CG）	Intervention in experimental group (*n*)	Interventio*n* in control group (*n*)	Outcomes
Li et al. [19]	China	BMD T-score ≤−2.5 SD	88	6 months	(65.1 ± 5.1/5.45 ± 1.45)/(65.5 ± 5.1/4.65 ± 1.42)	TCM exercises(30–40 min/day, 7 days /week) + basic treatment (*n* = 44）	Basic treatment (*n* = 44)	BBS, TUGT
Su and Deng [20]	China	BMD ＜80 mg/cm^2^	80	6 months	58.93 ± 4.01/59.12 ± 3.88	TCM exercises (45–60 min/time ,twice a day, 5 days/week) + basic treatment (*n* = 40)	Basic treatment (*n* = 40)	BBS, TUGT
Kuang [21]	China	BMD T-score ≤ −2.5 SD	82	6 months	(68.68 ± 3.22/1.79 ± 0.31)/(70.33 ± 3.34/1.58 ± 0.21)	TCM exercises (1 h/ time, twice a day, 7days/week) + basic treatment (*n* = 41)（	Basic treatment (*n* = 41)	BBS
Li et al. [22]	China	BMD T-score ≤−2.5 SD	111	6 months	63.25 ± 5.68/62.41 ± 6.09	TCM exercises (30–45 min/day, 2 days/week) + basic treatment (n = 56)	Basic treatment (*n* = 55)	BBS, TUGT
Shi et al. [23]	China	BMD T-score ≤−2.5 SD	64	6 months	63. 19 ± 6. 11/63. 59 ± 6. 35	TCM exercises (30–45 min/day, 3 days/week) + basic treatment (*n* = 32)	Basic treatment (*n* = 32)	BBS, TUGT
He et al. [24]	China	BMD T-score ≤−2.5 SD	43	1 month	59.08 ± 4.65/59.53 ± 5.40	PFMFs (40 min/day, 7 days/week) + basic treatment (*n* = 24)	Basic treatment (*n* = 19)	BBS, TUGT
Sun et al. [25]	China	BMD T-score ≤−2.5 SD	44	4 months	65.73 ± 2.46/69.58 ± 3.58	Strength training (60 min/day, 3 days/week) + basic treatment (*n* = 22)	Basic treatment (*n* = 22)	TUGT
Posch et al. [16]	Austria	BMD −2.5 SD ≤ T-score ≤ −1.0 SD	40	12 months	63.1 ± 4.53/62.78 ± 4.87	Balance and strength training(45–60 min/day, 2 days/week) (*n* = 20)	Basic treatment (*n* = 20)	TUGT
Teixeira et al. [17]	Brazil	BMD T-score ≤−2.5 SD	85	6 months	63.1 ± 4.53/62.78 ± 4.87	Balance and strength training (the specific time of each time is not available , 2 days/week) (*n* = 43)	Basic treatment (*n* = 20)	BBS, TUGT
Miko et al. [18]	Hungary	BMD T-score ≤−2.5 SD	100	12 months	69.33 ± 4.56/69.10 ± 5.30	Balance and strength training (25–35 min/day, 3 days/week) + basic treatment (*n* = 50)	Basic treatment (*n* = 50)	BBS, TUGT

## Data Availability

The Characteristics of the identified studies, study selection and data extraction used to support the findings of this study are included within the article (Table 1 and Figure 1). Previously reported RCTs were used to support this study and are available at PUMBED and CKNI. These prior studies are cited at relevant places within the text as references.
