# Corrigendum to “Effects of Nonpharmacological Interventions on Balance Function in Patients with Osteoporosis or Osteopenia: A Network Meta-Analysis of Randomized Controlled Trials”

**DOI:** 10.1155/2021/9892786

**Published:** 2021-06-05

**Authors:** Lu Zhu, Wenzhong Wu, Ming Chen, Daoming Xu, Huaning Xu, Lanying Liu, Jing Liu, Zequan Zhu

**Affiliations:** Affiliated Hospital of Nanjing University of Chinese Medicine, Nanjing 210029, Jiangsu, China

In the article titled “Effects of Nonpharmacological Interventions on Balance Function in Patients with Osteoporosis or Osteopenia: A Network Meta-Analysis of Randomized Controlled Trials” [1], the Acknowledgments section was omitted. The corrected section is given as follows.
